# HPVMD-C: a disease-based mutation database of human papillomavirus in China

**DOI:** 10.1093/database/baac018

**Published:** 2022-03-14

**Authors:** Zhenyu Yang, Wenjing Yi, Jin Tao, Xiaoqing Liu, Michael Q Zhang, Guiqian Chen, Qi Dai

**Affiliations:** College of Life Sciences, Zhejiang Sci-Tech University, Hangzhou 310018, China; College of Life Sciences, Zhejiang Sci-Tech University, Hangzhou 310018, China; College of Life Sciences, Zhejiang Sci-Tech University, Hangzhou 310018, China; College of Sciences, Hangzhou Dianzi University, Hangzhou 310018, China; Department of Biological Sciences, Center for Systems Biology, University of Texas at Dallas, Richardson, TX 75080, USA; Division of Bioinformatics, Center for Synthetic and Systems Biology, TNLIST, Tsinghua University, Beijing 100084, China; College of Life Sciences, Zhejiang Sci-Tech University, Hangzhou 310018, China; College of Life Sciences, Zhejiang Sci-Tech University, Hangzhou 310018, China; Department of Biological Sciences, Center for Systems Biology, University of Texas at Dallas, Richardson, TX 75080, USA

## Abstract

Human papillomavirus (HPV) can cause condyloma acuminatum and cervical cancer. Some mutations of these viruses are closely related to the persistent infection of cervical cancer and are ideal cancer vaccine targets. Several databases have been developed to collect HPV sequences, but no HPV mutation database has been published. This paper reports a Chinese HPV mutation database (HPVMD-C), which contains 149 HPV genotypes, 468 HPV mutations, 3409 protein sequences, 4727 domains and 236 epitopes. We analyzed the mutation distribution among HPV genotypes, domains and epitopes. We designed a visualization tool to display these mutations, domains and epitopes and provided more detailed information about the disease, region and related literature. We also proposed an HPV genotype prediction tool, which can predict HPV carcinogenic or non-carcinogenic risk genotypes. We expect that HPVMD-C will complement the existing database and provide valuable resources for HPV vaccine research and cervical cancer treatment. HPVMD-C is freely available at

**Database URL**: http://bioinfo.zstu.edu.cn/hpv.

## Introduction

Cervical cancer is one of the main causes to result in cancer incidence rate and mortality worldwide. More than 500 000 people are diagnosed with cervical cancer every year, and nearly 280 000 die from it (1, 2). Some studies have shown that human papillomavirus (HPV) is closely related to the incidence of cervical cancer, some genotypes of HPV can lead to abnormal growth of verrucous tissue (papilloma), and some HPVs are related to some cancers and precancerous diseases (3).

HPV is an icosahedral non-circular particle with small double-stranded circular DNA containing about 8000 nucleoside base pairs (4). It belongs to the papillomavirus family (papilloma, polyoma and simian vacuolation virus), and its diameter is about 55 nm (5). At least 150 HPV genotypes have been identified; some new genotypes will be defined if there is a significant difference between the new discoveries and the defined HPV genotypes (6, 7). Epidemiological studies have shown that genital HPV is closely related to cervical cancer, but not the other risk factors. These HPV genotypes were characterized by World Health Organization/International Agency for Research on Cancer as non-carcinogenic/unknown carcinogenicity, possibly carcinogenic and carcinogenic risk HPV genotypes (8). Non-carcinogenic/unknown carcinogenicity risk HPV genotypes are more closely related to low-grade lesions, while carcinogenic risk HPV genotypes are more closely related to high-grade cervical lesions and cancer. HPV16 and HPV18 accounted for 62.6% and 15.7% of cervical cancer, respectively. Therefore, identifying HPV genotypes with carcinogenic risk has become one of the important issues in the diagnosis and treatment of cervical cancer.

The distribution of HPV genotypes and intratype HPV genome variants in population and cervical cancer cases exhibits obvious regional characteristics (9). For example, HPV16 is widely distributed and has been divided into E (Europe), AA (Asia and America), Af-1 (Africa) and NA (North America) (10). In China, the total HPV infection rate is about 25%; the genotypes in the central region are HPV18, HPV33 and HPV58 (11), while the genotypes in the northern region are HPV16, HPV58, HPV18 and HPV33 (12). The carcinogenic risk of some mutation types in the same gene varies significantly in different countries and regions. For example, the HPV16 E6 variant prevalent in Europe and America is mainly G350 (L83V) (13), while in East Asia it is mainly G178 (D25E) (14). Some studies also confirmed that HPV16 E7 mutant has regional characteristics (15). In addition, mutations at some sites may make the virus more susceptible to induce carcinogenesis and increase the chances of re-infecting the host or fleeing the host immune system (16). For example, the carcinogenicity of HPV is mainly controlled by two proteins, E6 and E7 (17). These two proteins often produce intratypic variation (18). The mutation frequency of E6 in cervical cancer is 20–90% and E7 is 60–90% (19). Hu *et al.* confirmed that HPV16 E6 variant is related to human leukocyte antigen (HLA)-DRB1 and DQB1 alleles in Chinese young cervical cancer population (20). Qiu *et al.* found that some mutations of E6 gene will lead to amino acid changes, which may be more potentially carcinogenic (21). Studies in Japan have shown that the D25E mutation of HPV16 E6 is related to the DRB1*1502 allele of HLA II, which is considered to be an important mutation in invasive cancer and cervical intraepithelial neoplasia (22). Therefore, these gene variants of HPV increase the chance of re-infecting the host or fleeing the host immune system, which is important for finding the ideal target of cancer vaccine.

Several HPV-related databases have been proposed. For example, HPVdb (23) provides a large number of epitope data for T cell immunology and vaccinology. HPVbase (24) is a widely cross biomarker database, including data sets of virus integration, methylation patterns and abnormal expression of microRNA. PaVE (25) is a database of curated papillomavirus genomic sequences, accompanied by web-based sequence analysis tools. These databases are important for cervical cancer treatment and prognosis, but no HPV mutations database has been developed so far. In this paper, we proposed a Chinese HPV mutation database (HPVMD-C 1.0). It contains 149 HPV genotypes, 468 HPV mutations, 3409 protein sequences, 4727 domains and 236 epitopes (Supplementary Table S1). We discussed the distributions of HPV mutations among different HPV genotypes, various domains and epitopes and proposed some visualization techniques to display these mutations, domains, region and related literature. We also provided a blast search tool to facilitate user searching, as well as a HPV genotype prediction tool that can predict HPV genotypes from carcinogenic risk or non-carcinogenic risk.

## Materials and methods

### Disease-based mutation

One hundred and forty-nine HPV sequences and their mutations were collected through the search of National Center for Biotechnology Information (NCBI) (https://www.ncbi.nlm.nih.gov), China National Knowledge Infrastructure (CNKI) (https://www.cnki.net) and other public databases. There are 468 mutations covering 10 HPV genotypes (Supplementary Table S2) and 8 proteins (Supplementary Table S3), as well as the mutation types, location, sample disease and geographic information. In order to analyze the mutation distribution, the locations of these mutations are transformed into the whole genome. The reference sequences of 10 HPV genotypes are summarized in Supplemental Table S2.

### Domain detection

Some conserved domains of the HPV sequences were collected from the Conserved Domain Database (CDD, https://www.ncbi.nlm.nih.gov/cdd) which consists of a collection of well annotated multiple sequence alignment models for ancient domains and full length proteins and many domain models imported from external source databases, such as Pfam (http://ftp.ebi.ac.uk/pub/databases/Pfam), simple modular architecture research tool (SMART, http://smart.embl-heidelberg.de), clusters of orthologous genes (COG, https://www.ncbi.nlm.nih.gov/research/cog) and so on. For CDD, we set the parameter expect value as 0.01 and the default maximum number of hits is 500 to obtain the identification result. We used RADAR (26) to identify gapped approximate repeats and TMpred (27) to predict transmembrane region. Additional domains or motifs are from InterProScan (28), Motif Scan (29), SBASE (30), MOTIF Search, UniProt (31), PROSITE (32) and PROSITE Scan (33). All domains were then transformed into the whole genome, and the mutation sites were mapped and visualized. For HPV proteins, we used some online tools to identify domains in HPV protein sequences (Supplemental Table S4).

### Epitope data

Protein sequences that can be presented by one or more HLA alleles or recognized by T cells are considered T cell antigens. In addition, if peptides can stimulate the function of T cells, they are regarded as T cell epitopes. Peptides are considered HLA ligands if they have binding affinity with HLA molecules (23). Through the literature search in PubMed and Immune Epitope Database (34), a large number of experimentally verified T cell epitopes or HLA ligands involving 25 HPV genotypes were collected, and their sequences, HLA alleles, annotations and related references were manually organized.

### Carcinogenic risk HPV genotype prediction

In order to identify HPV genotypes at risk of carcinogenesis, a prediction algorithm was proposed based on HPV sequences. The prediction is divided into four steps: (i) 68 HPV genotypes, genome sequences and protein sequences are collected from NCBI, and eight data sets are constructed according to eight HPV proteins. (ii) Five hundred and twenty-two amino acid indexes were extracted and sorted from AAindex database (35). These indicators include hydrophobicity, pH value, solubility and other characteristics. An amino acid reduction algorithm was designed based on the physical and chemical properties of amino acids. (iii) Sequence features were extracted from the reduced protein sequence using the following six methods: PseAAC, Correlation, Kmer, Order, Position and RTCD. (iv) Support vector machine (SVM) was selected as the classifier to build the prediction model. In this work, we choose Gaussian radial basis function as kernel function. In order to evaluate the reliability, we selected the jack knife test to evaluate the performance of the proposed method and calculated the accuracy of each category and the overall accuracy as the standard performance measure. The detailed algorithm will be introduced in Supplementary section 9.

### BLAST

In order to find the mutation of the query sequence, the basic local alignment search tool (BLAST) for sequence similarity search is introduced into HPVMD-C (36). We downloaded BLAST v2. 6.0 (https://ftp.ncbi.nlm.nih.gov/blast/executables/blast+/2.6.0/) analysis tool to create a comparison library (36). Users can submit sequences by entering text or clicking the upload button to upload files. The submitted sequence must be in fast-all (FASTA) format in order to search using the BLAST algorithm (36).

### Database architecture

HPVMD-C system consists of three parts: client, server and database. The database is hosted on a web server running Apache (http://www.apache.org) on Windows 7 operating system. All data in the database are managed using MySQL (http://www.mysql.com). The web page of the client is generated using PHP (V5.6) scripting language (http://www.php.net). HPVMD-C has been successfully tested on Microsoft Internet Explorer 8, Firefox 60 and Google Chrome 66.

## Results and discussion

**Figure 1.**
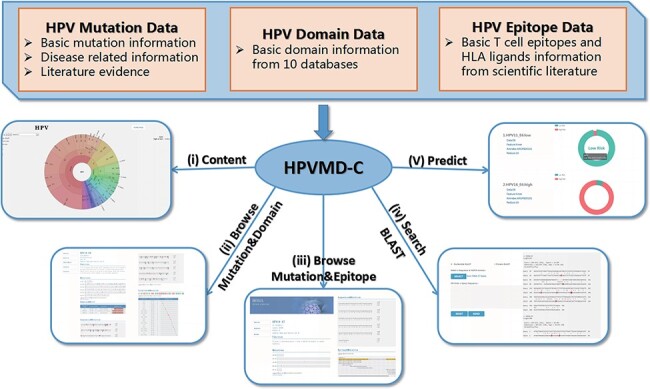
An overview of HPVMD-C 1.0: (i) Distribution of mutation content among the protein sequences, the domains and the epitopes; (ii) Distribution of the mutations among the domains; (iii) Distribution of the mutations among the epitopes; (iv) Mutation detection using BLAST; (v) Risk type prediction using the characteristics of amino acids and the SVM.

HPVMD-C contains 3409 protein sequences of 149 HPV genotypes, 468 HPV mutations and related clinical information, 4724 domains determined by ten online tools, and 236 epitope information collected through public databases (Supplementary Table S1). Using the mutation information of existing domain and epitope resources, HPVMD-C helps users to further study the relationship between pathogenic mutations and structurally conserved regions. Figure 1 outlines the data sources and unique functions of HPVMD-C. It consists of three data sets and five functions: (i) visualization of mutation distribution in sequence, domain and epitope; (ii) association analysis between mutation and domain; (iii) association analysis between mutation and epitope; (iv) search the mutation database using blast and (v) predicting unknown types of HPV proteins (e.g. carcinogenic risk or non-carcinogenic risk).

### Distribution of HPV mutations

**Figure 2.**
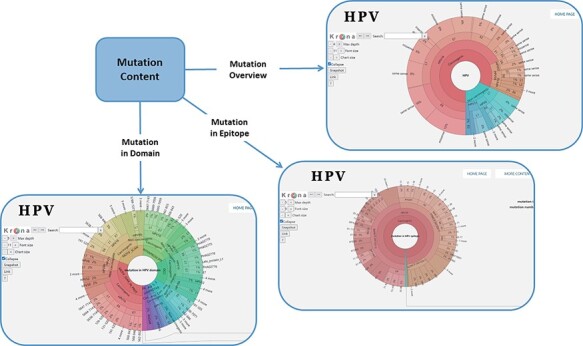
Distribution of mutation content among the whole sequences, the domains and the epitopes; the parameters on the left can adjust the display of the pie chart.

In order to facilitate the representation of mutation distribution, we proposed a mutation visualization module based on Krona (37, 38). In this module, the user can switch three pie charts by clicking the button. These pie charts show the mutation distribution of different HPV proteins, domains and epitopes (Figure 2). As for HPV genotypes with carcinogenic risk, HPV16 has the most mutations in E6 protein (32%) and L1 protein (24%). The percentage of missense mutations in E6 protein was 54% and contained a deletion mutation. As for the HPV domain, we found four known mutations (6%) in the 525–639 interval of HPV16 E7 obtained from UniprotKB, and L22F was located in this region (LXCXE motif). This motif can bind to the pocket structure (649–772) of PRB (retinoblastoma protein) and inhibit the effect of PRB (39, 40). Therefore, this mutation will affect the interaction between E7 and pRb (41), which will affect the regulation of the cell cycle. At the same time, there is a major E7 epitope during this period. In terms of epitope mutation, we found six mutations (4%) in the range of 21–42, such as L22F, N29S and N29H. They affect the immunogenicity of E7 protein.

### Distribution of HPV mutations in domains and epitopes

The distribution of mutations among conserved sites, CDD domains, UniProt domains and functional domains is shown in Supplementary Tables S5 and S6. It is easy to note that the mutations of E1 protein only involve carcinogenic risk genotype HPV16. Due to the long domain of E1 and the high coverage of protein, the mutation rate of E1 protein in CDD domain is as high as 100% (Supplementary Table S5). E2 mutations mainly involve in carcinogenic risk genotype HPV16 and non-carcinogenic risk genotype HPV2. E2 mutations are located in domains from CDD, InterProScan and Motif Scan, which are important sites affecting protein function. As for E5 protein, mutations occurred only in the carcinogenic risk genotypes HPV16 and HPV52. E6 and E7, as major oncoproteins, play a key role in carcinogenesis, and the proportion of mutations at their conserved sites is higher than that of other proteins (Supplementary Table S5). We found that there were almost no conserved sites in E6 and E7, which may be the reason for the short length of E6 and E7 proteins. For L1 and L2 proteins, they are primary capsid proteins and secondary capsid proteins. It is easy to note that L1 protein mutations are more than E6 protein mutations in non-carcinogenic risk HPV genotypes and carcinogenic risk HPV genotypes. As for domains, mutations in E6, E7, E2 and L1 proteins are more concentrated in conserved spatial domains (Supplementary Table S6), indicating that the conserved spatial structures of E6 and E7 are very important. More information on the distribution of mutations in the functional domains can be found in Supplementary section 8.

For the ‘mutation and epitope’ page, the user can select ‘mutation and epitope’ from the drop-down menu to access the list page of all relevant epitopes or enter the page from the scroll module. We collected the epitope data of 1169 sequences and showed the association between epitopes and mutations. We use a format that lists all epitopes and HLA ligands in the protein, with T cell epitopes or HLA ligands highlighted in yellow.

### Using BLAST to search mutation data

HPVMD-C provides users with a ‘BLAST’ search module. Users can input DNA or protein sequences or upload sequence files in FASTA format. After clicking ‘SEND’, the user will get an overwritten BLAST result page. Users can find the genotypes and proteins of similar sequences and obtain the published mutation information in all similar sequences, which will help to identify unknown mutations in new HPV sequences.

### The performance of carcinogenic risk HPV genotype prediction

To identify carcinogenic risk genotypes of HPV sequences, a prediction method was proposed and integrated into HPVMD-C. The proposed method was developed based on the characteristics of amino acids and SVM, its performance is represented in Supplementary Table S7. We further compared it with SVM based on the mismatch (42); SVM classifier based on the linear kernel (43); SVM based on the gap spectral kernel (Gap) (43), BLAST model (44) and integrated SVM (Ensemble) (44); and two text prediction methods based on AdaCost and naive Bayes (45). The accuracy of the proposed method is 98.4%, while that of integrated SVM is 94.12%, the SVM based on mismatched kernel is 92.70% and the SVM based on linear kernel is 90.28% and BLAST is 91.18%. For the text prediction method, the accuracy of AdaCost is 93.05%, while the accuracy of naive Bayes is 81.94%. The results indicate that the proposed method is more effective in predicting the carcinogenic risk HPV genotype. Users can submit a protein sequence and select the physicochemical properties of amino acids, number of reduced categories and different feature extraction methods, and then the prediction results can be obtained.

### HPV epitopes conservation analysis

HPVMD-C provides epitope protection analysis in the database, which is developed on the basis of multiple alignment using fast fourier transform (MAFFT, https://mafft.cbrc.jp/alignment) (46), a multiple sequence alignment program. It provides a series of alignment methods with excellent performance in accuracy and speed. In the ‘Epitope&Mutation’ module, each epitope can be linked to an epitope information page. Users can select an interest from this module and click the ‘check conservation’ button. It will then jump to the conservative analysis page, where the selected epitopes will be painted yellow.

### Sequence variation analysis of HPV16 E6 protein in Hong Kong

**Figure 3.**
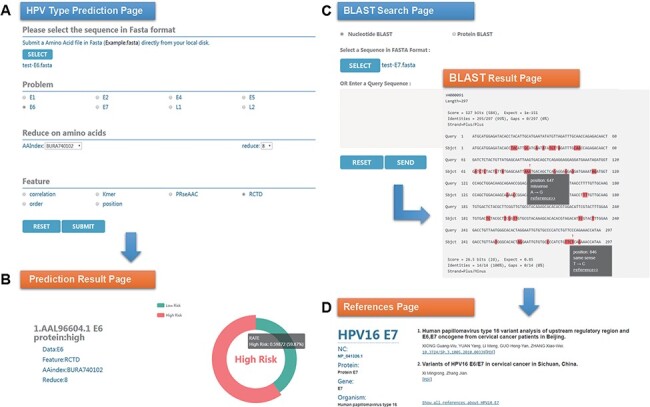
An example of HPVMD-C 1.0 usage: (A) Prediction page—Select optimal parameters: ‘E6’ data set, beta propensity characteristic index ‘BURA740102’, reduction number 8 and ‘RCTD’ feature; submit and get the result (B); (C) Blast search page and the result page—Use arrow to mark points that are different from the query sequence and highlight the mutation about this sequence in red; using mouse to slide over a mutation will pop up a detail box; references result page (D) will be displayed by clicking the references link.

Chan *et al.* (47) analyzed the sequence variation of HPV16 E6 protein (AAL96604.1) in Hong Kong women with cervical cancer and found that the 350G variant is rare in Asia. Two mutations in E7 protein, nt 647 A→G and nt 846T→C, have high mutation rates in Asian isolates and are not common in European isolates. To evaluate the performance of HPVMD-C, we first predicted the HPV genotype of E6 protein with the selected parameters in Figure 3A. Figure 3B indicates that the incidence of the carcinogenic risk type is close to 60%, which is consistent with the results of Muñoz’s paper (48). We then analyzed its mutations of DNA sequence (AF486326.1) corresponding to E7 protein with the help of BLAST in HPVMD-C. Figure 3C shows that the same mutations are reported at both Sites 647 and 846. The mutation at 647 is a missense mutation, and the mutation at 846 is the same-sense mutation. By clicking the ‘reference’ link at point 647, we obtained 2 papers related to the mutation and 33 papers related to E7 protein, as shown in Figure 3D.

**Figure 4.**
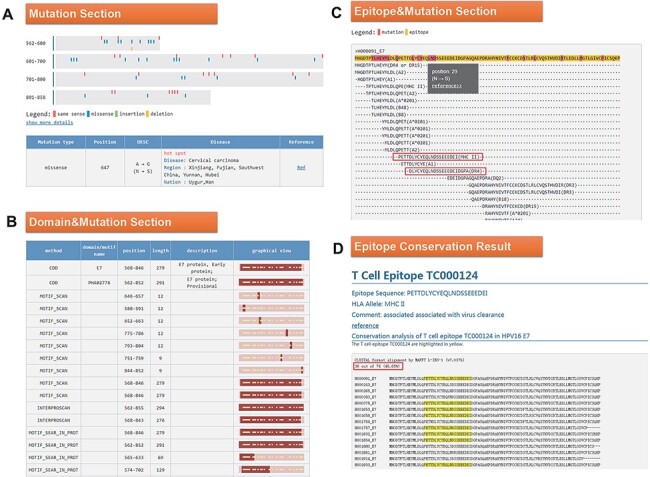
(A) Mutation section in ‘Detail’ page: Use visualization techniques to show the position and type of mutations; related diseases, races, regional information and literature links are also listed; (B) Domain section in detail page: In the graphical view, bar represents the entire sequence, dark part represents the domain or motif and dots represent the mutation; (C) Epitope section in detail page: The second line shows the entire sequence, light part highlights the epitope and dark part highlights the mutation; inside two boxes are the epitopes containing N29S; (D) Epitope conservation result page: The query epitope TC000124 are highlighted; the conservation rate is 48.65%.

Click the ID number (H000091) on the BLAST result page to enter the ‘Detail’ page, as shown in Figure 4. In the mutation part, we found that the 647 mutation is very common in Southwest China, Uyghur and Han (Figure 4A). Among the 34 domains displayed in the domain and mutation section, 16 domains contain this mutation (Figure 4B). Since the mutation at Position 647 leads to the change of amino acid at Position 29, we want to know whether this mutation will affect the immunogenicity of the epitope. In the last part of the results page, we found two T cell epitopes containing this mutation (Figure 4C). Click on the first epitope and check the conservation of this epitope by the ‘check conservation’ button; Figure 4D shows that Tc000124 epitope was conserved in 36 of 74 (48.65% conserved) HPV16 E7 complete sequences. Ten protein sequences mutated at this site (N29S, etc.) lead to immune escape.

### Comparison with other HPV databases

Some databases have been developed specifically for HPV. For example, HPVdb (23) database provides HPV antigens and peptides data for T cell immunology and vaccinology. It provides several methods to search for antigens and immune epitopes, as well as the visualization of T cell epitopes or HLA ligands. It has complete sequence, UniProt status, sequence status, list of T cell epitopes and HLA ligands of antigens, as well as references. HLA binding prediction tools are also provided in this database. PaVE (25) is a database of curated papillomavirus genomic sequences, accompanied by web-based sequence analysis tools. hpvPDB is the human papillomavirus proteome database (49), which includes the details of sequencing submission, disease type, molecular weight, nucleotide composition, gene number, etc. It also provides a phylogenetic analysis tool.

Although the above two databases provide researchers with important sequence information and related immune information, they do not include HPV mutation data, which is an ideal target for cancer vaccine. HPVMD-C contains 149 HPV types, 468 HPV mutations, 3409 protein sequences, 4727 domains and 236 epitopes. It has visualization technology to display these mutations, domains and epitopes and provide more detailed information about disease, region, race and related literature. It also provides a BLAST to facilitate user search and an HPV genotype prediction tool, which can predict the carcinogenic risk or non-carcinogenic risk genotype of unknown HPV.

## Conclusions and future perspectives

HPV mutation and epidemiological data of cervical cancer in China will play an important role in the development of cervical cancer vaccine. HPVMD-C not only provides convenient browsing and search functions, mutation and domain or epitope combination analysis but also includes a tool to predict HPV genotypes. In order to make the database as comprehensive as possible, we collected a large number of identified HPV sequences, mutations and epitopes in China and used various online tools to identify sequence domains. We first combined domains, secondary structures and epitopes with mutations to find some regions that may lead to functional changes and may affect immunogenicity. We introduced some visualization techniques to display these mutations, domains and epitopes and provided more detailed information about disease, region, race and related literature. We also provided a BLAST to facilitate user search and an HPV genotype prediction tool to predict HPV carcinogenic risk or non-carcinogenic risk genotypes. In the future, since more data sets are available, we will regularly update HPVMD-C and add HPV mutations and related domains in other HPV genotypes to expand the database. We expect that HPVMD-C will supplement the existing database and provide valuable resources for HPV vaccine research and cervical cancer treatment.

## Supplementary Material

baac018_Supp
