# Supplementary material for: HPVMD-C: a disease-based mutation database of human papillomavirus in China
Source: Database (Oxford). 2022 Mar 14;2022:baac018. doi: 10.1093/database/baac018 (PMC9216535; doi:10.1093/database/baac018)
Supplement: baac018_Supp [file baac018_supp.zip › HPVDB-supplementary 0301 ...docx]

Supplementary Information: HPVMD-C: a disease-based mutation database of human papillomavirus in China

Zhenyu Yang1, Wenjing Yi1, Jin Tao1, Xiaoqing Liu3, Michael Q Zhang2,5, Guiqian Chen1, *, Qi Dai1,2,*

1College of Life Sciences, Zhejiang Sci-Tech University, Hangzhou 310018, China.

2 Department of Biological Sciences, Center for Systems Biology, University of Texas at Dallas, Richardson, TX 75080, USA.

3 College of Sciences, Hangzhou Dianzi University, Hangzhou 310018, China.

4 College of Sciences, Zhejiang Sci-Tech University, Hangzhou 310018, China.

5 Division of Bioinformatics, Center for Synthetic and Systems Biology, TNLIST, Tsinghua University, Beijing, 100084, China.

1. **Table S1. Various datasets in HPVMD-C**

| Datasets | Size | HPV genotypes(n) | Source |
| --- | --- | --- | --- |
| Protein sequence | 3409 | 149 | NCBI and other databases https://www.ncbi.nlm.nih.gov/protein |
| Mutation | 468 | 10 | Published literature |
| Epitope | 236 | 25 | PubMed and Immune Epitope Database http://www.iedb.org |
| Domain | 4724 | 10 | Identified by 10 online tools |
| Structure | 242 | 4 | Uniprot database https://www.uniprot.org/uniprot |

1. **Table S2.** **Genotypes, genome ID, mutation number, and proteins associated with HPV mutations**

| **Risk Type** | **Genotype** | **Genome ID** | **Mutations**(n) | **Proteins** |
| --- | --- | --- | --- | --- |
| Carcinogenic | HPV16 | NC_001526.2 | 317 | E1、E2、E5、E6、E7、L1、L2 |
| Carcinogenic | HPV18 | AY262282.1 | 12 | E6、E7、L1、L2 |
| Carcinogenic | HPV58 | D90400.1 | 20 | E6、E7、L1 |
| Carcinogenic | HPV52 | X74481.1 | 21 | E6、E7、L1 |
| Non-carcinogenic | HPV2 | NC_001352.1 | 7 | E2 |
| Carcinogenic | HPV45 | X74479.1 | 7 | E6 |
| Carcinogenic | HPV59 | X77858.1 | 4 | E6 |
| Non-carcinogenic | HPV6b | NC_001355.1 | 30 | L1 |
| Non-carcinogenic | HPV11 | M14119.1 | 23 | E6、E7、L1 |
| Non-carcinogenic | HPV6 | AF092932.1 | 24 | E6、E7、L1、L2 |

Carcinogenic risk type is more common than non-carcinogenic risk type, and HPV16 has more mutations.

1. **Table S3. Distribution of mutations in proteins of carcinogenic risk or non-carcinogenic risk HPV genotypes**

| **Proteins** | **Mutations(n)** | **Non-carcinogenic risk type(n)** | **Carcinogenic risk type(n)** | **HPV** **Genotypes** |
| --- | --- | --- | --- | --- |
| E1 | 13 | 0 | 13 | HPV16 |
| E2 | 43 | 7 | 36 | HPV2、HPV16 |
| E5 | 11 | 0 | 11 | HPV16 |
| E6 | 142 | 11 | 131 | HPV6、HPV11、HPV16、HPV18、HPV45、HPV52、HPV58、HPV59 |
| E7 | 91 | 5 | 86 | HPV6、HPV11、HPV16、HPV18、HPV52、HPV58 |
| L1 | 140 | 56 | 84 | HPV6b、HPV6、HPV11、HPV16、HPV18、HPV52、HPV58 |
| L2 | 25 | 5 | 20 | HPV6、HPV16、HPV18 |

E4 protein is encoded by E2 fragment, so we only need to analyze the mutation data of E2 protein. The mutation numbers of E6, E7 and L1 proteins are 142, 91 and 140 respectively, which are much higher than those of other proteins. Mutations in non-carcinogenic risk HPV genotypes (HPV6, HPV6b and HPV11) were mainly detected in the tissues of patients with acute condyloma. HPV18, HPV58, and HPV58 mutations are commonly detected in patients with cervical cancer.

1. **Table S4. Different functional domains and their online tools**

| Tools Websites | | Domains(n) | Result type |
| --- | --- | --- | --- |
| CDD | https://www.ncbi.nlm.nih.gov/Structure/cdd/wrpsb.cgi | 172 | Protein Domain |
| InterProScan | http://www.ebi.ac.uk/interpro/search/sequence-search | 330 |
| PROSITE Scan | https://prosite.expasy.org/scanprosite/ | 1315 |
| SBASE | http://pongor.itk.ppke.hu/protein/sbase.html#/sbase_form | 116 |
| UniProt | https://www.uniprot.org/uniprot/ | 115 |
| PROSITE | https://prosite.expasy.org/ | 10 |
| RADAR | https://www.ebi.ac.uk/Tools/pfa/radar/ | 228 | Repeat regions |
| TMpred | https://embnet.vital-it.ch/software/TMPRED_form.html | 265 | Membrane-spanning regions |
| Motif Scan | https://myhits.isb-sib.ch/cgi-bin/motif_scan | 1610 | Known motifs |
| MOTIF Search | https://www.genome.jp/tools/motif/ | 563 |

1. **Table S5. The percentage of mutations in protein conserved sites, CDD domains, UniProt domains, and the proportion of functional domains in protein sequences**

| **Protein** | **Mutations(n)** | **Percentage of mutations in conserved sites** | **Percentage of conserved sites in proteins** | **Percentage of mutations in**  **CDD domains** | **Percentage of CDD domains in proteins** | **Percentage of mutations in UniprotKB domains** | **Percentage of UniprotKB domain in proteins** |
| --- | --- | --- | --- | --- | --- | --- | --- |
| E1 | 13 | 2.30% | 21.43% | 100.00% | 100.00% | 35.71% | 49.46% |
| E2 | 41 | 18.30% | 19.51% | 60.98% | 73.02% | 9.76% | 9.21% |
| E5 | 11 | 73.33% | 0.00% | 90.91% | 59.04% | -- | -- |
| E6 | 134 | 100.00% | 5.97% | 87.31% | 88.38% | 36.57% | 49.08% |
| E7 | 86 | 100.00% | 13.95% | 98.84% | 99.16% | 73.26% | 81.93% |
| L1 | 166 | 28.23% | 22.89% | 95.78% | 93.38% | 0.00% | 2.27% |
| L2 | 24 | 6.86% | 20.83% | 100.00% | 97.78% | 0.00% | 4.55% |

HPV encodes 8 proteins, only 7 proteins are analyzed. We analyzed the mutation of E4 protein in E2 protein because E4 protein is encoded in a fragment of the E2 coding region.

Mutations of E1 protein in HPVDB-C database only involve the carcinogenic risk HPV genotypes HPV16. Due to the long domain of E1 and the high coverage of protein, the mutation rate of E1 protein in the CDD domain is as high as 100%. The mutation of E2 mainly involves the carcinogenic risk HPV genotypes HPV16 and the non-carcinogenic risk HPV genotypesHPV2. E2 mutations are all located in CDD, InterProScan, and MotifScan domains, which are associated with the important sites and affect the function of proteins. For E5 protein, the mutations occurred only in the carcinogenic risk HPV genotypes HPV16 and HPV52. E6 and E7 play a key role in carcinogenesis as main cancer proteins, there are many mutations in these proteins. The percentage of mutations and conserved sites is higher than that of others, even more than 100%. Comparing the conserved site percentage, we found that E6 and E7 were less than the others, which may be the reason for the short length of E6 and E7 proteins. For L1 and L2 proteins, they are major capsid proteins and secondary capsid proteins. It is easy to find that L1 protein has more mutations than other proteins.

1. **Table S6. The proportion of mutations in structurally conserved regions and the proportion of conserved regions in protein structures**

| **Protein** | **Proposition of mutations**  **in conserved regions** | **Proposition of conserved regions**  **in proteins** |
| --- | --- | --- |
| E1 | 0.00% | 69.11% |
| E2 | 51.22% | 77.09% |
| E6 | 38.81% | 50.61% |
| E7 | 26.74% | 22.48% |
| L1 | 81.93% | 81.08% |
| L2 | 0.00% | 3.80% |

1. **Table S7. Prediction accuracy of the proposed method on various proteins**

| **Protein datasets** | **Totality accuracy** | **Accuracy of** **carcinogenic risk type** | **Accuracy of non-carcinogenic risk type** |
| --- | --- | --- | --- |
| **E1** | 0.953 | 0.861 | 0.984 |
| **E2** | 0.917 | 0.764 | 0.969 |
| **E4** | 0.892 | 0.647 | 0.975 |
| **E5** | 0.871 | 0.917 | 0.815 |
| **E6** | 0.931 | 0.826 | 0.966 |
| **E7** | 0.750 | 0.010 | 0.994 |
| **L1** | 0.984 | 0.856 | 1.000 |
| **L2** | 0.963 | 0.931 | 0.974 |

1. **Association analysis between HPV mutation and functional structure region**

**E1 protein**

E1 protein has three main functional domains. 13 mutations were found in HPV16 E1, including 4 in the N-terminal domain, 4 in the C-terminal helicase domain, and 5 in the DNA binding domain. DNA 1950-2230 expression product has good immunogenicity. This region is located in the C-terminal domain, and its corresponding protein fragments are highly conserved. The results show that 4 mutations are located at highly conserved sites in the C-terminal helicase domain.

**E2 protein**

As a specific DNA binding protein, E2 plays an important role in viral mRNA translation and DNA replication, especially in regulating the transcription of E6 and E7 proteins. The destruction, low expression, or deletion of genes in E2 protein will terminate the life cycle of the HPV virus. At the same time, the deletion of E2 protein leads to the imbalance and loss of inhibition of E6 and E7 proteins, which means the high expression of E6 and E7 proteins. The results show that there are 7 mutations in the structural site of HPV16 E2 protein, one at the N-terminal DNA binding site, 4 in the hinge region, and 2 inhibited the expression of E6 and E7 at the C-terminal. Studies have shown that the mutation of 3697 has the same effect as the deletion of the whole N-terminal(1). HPV16 has 37 mutations, 13 in the N-terminal, 8 in the C-terminal, and the other 13 mutations in E4 protein. The N-terminal 3159 mutation can change the immune function of the protein, and the C-terminal 3684 mutation is close to the helix. 3410 mutation of E4 protein may be related to the occurrence and development of invasive cervical cancer.

**E5 protein**

E5 protein contains two main domains: hydrophobic amino-terminal domain and hydrophilic carboxyl-terminal domain. This protein not only increases host cell immune escape, regulates the cell cycle, but also promotes cell transformation and increases apoptosis. In addition, we have discovered transmembrane conserved regions and hydrophobic residues. We found that two mutations 3979 and 3883 fall in the transmembrane conserved region, and a mutation 4041 occurs in the positive selection pressure site, which may change the function of E5 protein. Other mutations are located near these three special mutations.

**E6 protein**

E6 protein involves a large number of genotypes. We analyzed non-carcinogenic risk HPV and carcinogenic risk HPV genotypes, respectively. Non-carcinogenic risk HPV genotypes includes HPV6, HPV6b and HPV11. We analyzed HPV6 and HPV6b together because they have only three differences. Only one encoded amino acid is different, and none of the three amino acids belong to conservative sites.

The non-carcinogenic risk E6 protein has only one nuclear localization signal (NLS) 3 or a functionally similar fragment of NLS3 and two zinc finger domains. HPV6b and HPV11 have six important amino acids (Table S8), there were 7 mutations in the non-carcinogenic risk types, 5 of which are in the NLS3 or zinc finger domain, but none of them in the Table S8.

**Table S8. Six important amino acids in HPV6b and HPV11 E6 proteins**

| **Mutation position** | **Starting point of the whole gene** | **Endpoint of the whole gene** |
| --- | --- | --- |
| 56 | 267 | 269 |
| 64 | 291 | 293 |
| 78 | 333 | 335 |
| 111 | 432 | 434 |
| 133 | 498 | 500 |
| 137 | 510 | 512 |

In the carcinogenic risk HPV genotype , E6 protein of HPV16 has 91 mutations, 45 of which fall within the zinc finger domain or NLS. From the results of spatial structure superposition, it can be seen that zinc finger domain is spatially conserved in both non-carcinogenic risk and carcinogenic risk HPV genotypes , where E6 protein binds to multiple target proteins of the host cell. There are three mutations in E6 protein of HPV18. 218 mutation is located in zinc finger 1 region, 485 mutation is located in zinc finger 2 region, and 549 mutation is located in the short terminal region of linear marker protein. E6 protein of HPV45 has 8 mutations, 5 mutations in the zinc finger or nuclear localization signals, and the other are in the B cell epitope region. The homology of HPV45 and HPV18 is very high. 482 mutation of E6 protein of HPV45 is very similar to that of HPV18. 549 mutation of HPV18 E6 (the 445th base in E6 gene) and 259 (the 158th base in E6 gene) and 497(the 396th base in E6 gene) mutations of HPV45 E6 are internal transformations of two homologous regions (2). HPV52, HPV58 and HPV59 do not have a clear NLS area.Among them, HPV52 mainly causes moderate to high lesions of cervical epithelium and rarely causes cervical cancer. There are 13 mutations in the E6 protein of HPV52, 10 of which are from Sichuan and 3 in Guangdong. One of the 10 mutations from Sichuan is in the zinc finger domain 1 region, and the others are located inside the unknown functional region. Before the mutation at position 379, the amino acid K is located at the end of the helix, and after the mutation, K is located outside the alpha helix. For E6 protein of HPV58, we collected 5 mutations, and these mutations are all within the conserved region between the zinc finger domain 1 region and the zinc finger domain 2 region. E6 protein of HPV59 has four mutations located in a structurally conserved region. However, no reports of the zinc finger domain and NLS have been found in HPV59.

In the N-terminal of E6 protein, regions such as 2-12, 78-82, 111-115, 118-132, etc. may be bound to p53. Any amino acid mutation at these positions may alter the ability of E6 to bind to p53. There are 11 mutations in E6 protein of non-carcinogenic risk HPV, 9 of which are in the p53 protein binding region or antigenic determinant region, and 49 of 91 mutations fall in these regions in the carcinogenic risk types. Therefore, the proportion of non-carcinogenic risk type mutations in the p53 protein binding region or antigenic determinant region is higher.

**E7 protein**

E7 protein consists of three CR regions: CR1 (1-15), CR2 and CR3 (65-69). The N-terminal CR1 is required for cell transformation and pRb degradation but does not directly bind to pRb. CR2 contains the conserved pRb binding sequence LxCxE (22-26) and two protein kinase phosphorylation sites (31, 32)(3). CR3 consists of two C-x-x-C functional domains.

As for the carcinogenic risk type, E7 protein structure of HPV16 is highly conserved so that amino acid changes cannot be tolerated. We found that 23 of 64 mutations are located inside the CR region. The coverage of three CR regions in the protein is 27.55%. The proportion of these mutations falling within E7 protein domains is as high as 93%. Among 23 mutations, 14 are located in CR1 and 4 in the LxCxE region. The remaining five mutations are in the CR3 region, which contains two zinc finger domains and some CTL sites. In HPV18, there are only two mutations in E7 protein, one is within the alpha-helix region and the other is within the randomly folded coil region. The HPV58 E7 protein has 8 mutations, 760 mutation is located in the protein folding corner region, while the two synonymous mutations 726 and 744 are located in the beta-sheet region of the protein. Among the carcinogenic risk E7 proteins, we find 5 mutations, only one of them falls within the CR2.

**L1 protein**

L1 protein mutations are mainly distributed in four regions: SR1 (5911-6140), SR2 (6217-6273), SR3 (6540-6661) and SR4 (7062-7250) in HPV6 and HPV6b. However, L1 antigen intensity does not change due to the mutations (4). In L1 protein of HPV16, 6902 mutation (insert three bases ATC), 6954 (deleted three bases GAT) and 6178 will cause changes in B lymphocyte epitopes and affect the ability of antibodies to recognize and neutralize antibodies. Studies have shown that the mutations at positions 83-97 affect protein expression and even affect the folding of virus-like particles in L1 form, while 6240 mutation reduces VLP formation.

1. **Carcinogenic risk HPV types prediction**
2. **Dataset construction**

There are eight open reading frames that encode early and late genes of the HPVs [11]. The early and late genes have polyA signal 1 and polyA signal 2. The produce of the late genes are L1 and L2 proteins which affect the viral capsid structure [5], while early genes are transformed into E1- E7 proteins. We constructed seven protein databases of the HPVs whose sequences are downloaded from the Los Alamos National Laboratory (LANL). Each protein has 72 HPV types. If a certain type of protein lacks the sequences of HPVs, we downloaded the missing sequence from the National Biotechnology Information Center. Since E4 protein cannot be found in the National Biotechnology Information Center, its total number is 71. According to HPV compendium, seventeen HPV types are classified as carcinogenic risk HPV genotypes, and the remain are possibly carcinogenic risk HPV genotypes [6].

1. **Amino acid reduction**

20 amino acids have subtle differences, but some of them have similar basic structures and functions. AAindex is a database of physical and biochemical indicators of amino acids established by Tomii and Kanehisa [7]. It mainly includes three parts: AAindex 1, AAindex 2 and AAindex 3. AAindex 1 is a database that describes the physicochemical and biological properties of amino acids. AAindex 2 is the matrix of amino acid mutation, and AAindex 3 is the protein contact potential statistics. These data are from published articles. We mainly used AAindex 1 to calculate the correlation coefficient as the distance between the two indicators. AAindex 1 currently contains 544 indexes, and this article selected 522 indexes. These 522 characteristics are further divided into 7 categories (A): Alpha and turn propensities, (B): Beta propensity, (C): Composition, (H): Hydrophobicity, (P): Physicochemical properties, and (O): Other properties [7].

The Euclidean distance between two amino acids is calculated based on the physical and biochemical indicators of the amino acids in AAindex database, and then the distance matrix is obtained. Then, 20 amino acids are reduced into given classes (RedAAs) by using unweighted pair group method with arithmetic mean (UPGMA).

1. **Feature extraction**

**K-mer**

Protein sequences and peptides can be seen as a collection of symbols, and their characteristics can be analyzed by the frequency of their small fragments. *k*-mers are k consecutive characters in reduced proteins, and a sliding window of length *m* can be used to calculate their frequencies [8], moving from position 1 to *m-k+1* with one base at a time. It allows the overlaps of the *k*-mers and is calculated as:

|  |  |  |
| --- | --- | --- |

where is occurrence number of the *k*-mer, and is *k*-mer set of the RedAAs.

**RCTD**

“Composition (C)”, “Transition (T)”, and “Distribution (D)” are three descriptors of RedAAs, which are defined as follows [9]. Composition can be regarded as a single monomer of the reduced sequence, and the sequence components are described by calculating the percentage of each RedAA. Transition is used as the conversion of RedAA *I* and *A* by calculating the frequency of *I* followed *A*

|  |  |  |
| --- | --- | --- |

and are the “*IA*” number and “*AI*” numbers in the reduced sequence with length *N.* Distribution describes the RedAA distribution in the reduced sequence, including the specified coding categories: 25%, 50%, 75% and 100%.

**PRseAAC**

PRseAAC is a kind of widely used pseudo-reduced AA compositions (PRseAAC) [10]. It was proposed by Kuo-chen Chou, which is defined as follows:

|  |  |
| --- | --- |
|  |  |

where is the RedAA frequency, *w* is the weighting factor. is calculated as:

|  |  |
| --- | --- |
|  |  |
|  |  |

is the RedAAs’ property*,* and *R* is the RedAA size.

**Correlation**

Moran autocorrelation is based on the characteristics of correlation, which describes the correlation among the RedAAs [11]. The RedAA *M* can be calculated as:

|  |  |  |
| --- | --- | --- |

where denotes the RedAA property at position *i* of the sequence, *d* is the autocorrelation lag, and *N* is the sequence length.

**Order**

Order reflects the physical and chemical interaction among the RedAA pairs [12]. The quasi-sequence score of the RedAA is defined

|  |  |  |
| --- | --- | --- |

where is the RedAA frequency*,* and *w* denotes a weighting factor.

**Position**

Position feature represents the distribution of RedAA positions of protein sequences based on the coefficient of variations [13]. First, we converted the protein sequence into a digital sequence *N*(RedAA), and calculated the probabilities of the separation distance between two adjacent RedAAs. The mean and variance are defined:

|  |  |  |
| --- | --- | --- |
|  |  |  |

We then calculated the positional information

|  |  |  |
| --- | --- | --- |

is the reciprocal of the coefficient of variation (C.V) which compares the degree of change between two datasets, even if there are large differences between their means.

1. **Prediction algorithm**

is an HPV label set,  is from high-risk type, and is from low-risk type. We used to represent theth features of the RedAA modes of the th HPV sample, where . All of the features of the RedAA modes for all HPV samples are denoted as

|  |  |  |
| --- | --- | --- |

We used support vector machine (SVM) to predict HPV type, which is expressed as follows:

|  |  |  |
| --- | --- | --- |
|  |  |  |

where is a linear combination of a set of nonlinear data conversion:

|  |  |  |
| --- | --- | --- |

denotes a bias term, and denotes some regularization parameters and is the training error. The above problem can be expressed:

|  |  |  |
| --- | --- | --- |
|  |  |  |

Here, the Gaussian kernel function is used to calculate instead of and . The separation problem can be expressed

|  |  |  |
| --- | --- | --- |
|  |  |  |

The training model can predict the risk type of the test sample according to the following formula:

indicates that the sample belongs to high-risk type; otherwise, it belongs to low-risk type. In order to obtain better model, we used a simple grid search strategy based on 10-fold cross-validation to find the optimal model for each data set.

References

1. Gao, C., et al.(2011) [Mutations in various functional domains of HPV2 E2 protein inhibit the transcriptional depression activities]. Zhonghua shi yan he lin chuang bing du xue za zhi = Zhonghua shiyan he linchuang bingduxue zazhi = Chinese journal of experimental and clinical virology, 25, 164-166.

2. Wu, E.Q., et al.(2006) Analysis of E6 variants of human papillomavirus type 18, 45 isolated from cervical cancer patients in Sichuan Province. Chemical Journal of Chinese Universities, 27, 263-267.

3. Boulet, G., et al.(2007) Human papillomavirus: E6 and E7 oncogenes. International Journal of Biochemistry & Cell Biology, 39, 2006-2011.

4. Perkins, S.M., et al.(1999) Intratype sequence variation among clinical isolates of the human papillomavirus type 6 L1 ORF: clustering of mutations and identification of a frequent amino acid sequence variant. Journal of General Virology, 80 ( Pt 4), 1025-1033.

5. Longworth MS, Laimins LA. (2004) Pathogenesis of human papillomaviruses in differentiating epithelia, Microbiol. Mol. Biol. Rev. 68: 362-372.

6. Kim S, Kim J, Zhang BT. (2009) Ensembled support vector machines for human papillomavirus risk type prediction from protein secondary structures, Computers in Biology and Medicine. 39: 187-193.

7. Tomii K, Kanehisa M. (1996) Analysis of amino acid indices and mutation matrices for sequence comparison and structure prediction of proteins, Protein Eng. 9(1): 27-36.

8. Dai Q, Li Y, Liu XQ, Yao YH, Cao YJ, He P. (2013) Comparison study on statistical features of predicted secondary structures for protein structural class prediction: From content to position, BMC Bioinformatics. 14: 152.

9. Cui J, Han LY, Lin HH, Zhang HL, Tang ZQ, Zheng CJ, Cao ZW, Chen YZ. (2007) Prediction of MHC-Binding Peptides of Flexible Lengths from Sequence-Derived Structural and Physicochemical Properties, Mol Immunol. 44: 866-877.

10. Shen HB, Chou KC. (2007) Using ensemble classifier to identify membrane protein types, Amino Acids. 32(4): 483-8.

11. Horne DS. (1988) Prediction of protein helix content from an autocorrelation analysis of sequence hydrophobicities, Biopolymers. 27: 451-477.

12. Chou KC. (2000) Prediction of protein subcellular locations by incorporating quasi-sequence-order effect, Biochem Biophys Res Commun. 278:477-483.

13. Zhang SL, Liang YY, Yuan XG, (2014) Improving the prediction accuracy of protein structural class: Approached with alternating word frequency and normalized Lempel-Ziv complexity, J Theor Biol. 341: 71-77.
